# Grasping Performance Analysis and Comparison of Multi-Chamber Ring-Shaped Soft Grippers

**DOI:** 10.3390/biomimetics8040337

**Published:** 2023-07-31

**Authors:** Dan Wang, Xiaojun Wu

**Affiliations:** School of Mechanical and Electrical Engineering, Xi’an University of Architecture and Technology, Xi’an 710055, China; danwang@xauat.edu.cn

**Keywords:** ring-shaped soft gripper, grasping performance, finite element method (FEM), load capacity

## Abstract

Biologically inspired pneumatic ring-shaped soft grippers have been extensively studied in the field of soft robotics. However, the effect of the number of air chambers on the grasping performance (grasping range and load capacity) of ring-shaped soft grippers has not been studied. In this article, we propose three ring-shaped soft grippers with the same area of inner walls of air chambers and different numbers of air chambers (two-chamber, three-chamber, and four-chamber) for analyzing and comparing their grasping performance. Finite element method (FEM) models and experimental measurements are conducted to compare the deformation of the inner walls of the three ring-shaped soft grippers, the results indicate that the grasping range of the three-chamber ring-shaped soft gripper is larger than that of the two-chamber ring-shaped soft gripper and the four-chamber ring-shaped soft gripper. Then we choose the three-chamber ring-shaped soft gripper to study the relationship between contact force and air pressure by FEM models and experimental measurements. Several groups of experiments are constructed to compare the load capacity of the three ring-shaped soft grippers, the results indicate that the load capacity of the three-chamber ring-shaped soft gripper is higher than that of the two-chamber ring-shaped soft gripper and the four-chamber ring-shaped soft gripper. The above results reveal that the grasping performance of the three-chamber ring-shaped soft gripper is better than that of other two ring-shaped soft grippers. Furthermore, the application experiments indicate that the three ring-shaped soft grippers can grasp various objects with different weights, material properties, and shapes. This study provides a new idea for investigating ring-shaped soft grippers.

## 1. Introduction

Bionics is a comprehensive fringe discipline, which provides new design concepts and creates new products for medical, military, architecture, industry, and other fields by imitating the structure, function, and behavior of organisms [1]. Bionics has provided a source of inspiration for the design and study of soft robots, such as an untethered quadrupedal soft robot [2], an acoustically controlled soft robotic fish [3], and a bionic flower for robotic grippers [4]. Compared with traditional rigid robots, soft robots have higher flexibility, safety, environmental adaptability, and other characteristics; therefore, soft robots have been widely considered by researchers.

Soft grippers are an important branch of the soft robot field. Soft grippers made of flexible materials cannot only adaptively grasp or manipulate objects with complex shapes, variable shapes, and fragile material, but can also ensure the safety of cooperation with people and the environment. Therefore, soft grippers have received widespread attention and continued research [5,6,7,8,9,10,11].

At present, some methods used to drive soft grippers include tendon drive [12,13], pneumatic drive [14,15,16,17], shape memory alloy drive [18,19], electroactive polymer drive [20,21,22], etc. Pneumatic drive has taken up an important position in the field of soft grippers due to its characteristics of large driving force, fast response speed, no pollution, and strong environmental adaptability. In order to improve the grasping performance of pneumatic soft grippers, researchers have developed soft grippers with different structures. Pneumatic soft grippers have been divided into multi-finger structure [23,24,25,26,27,28,29,30], closed structure [31,32,33,34,35], and ring-shaped structure [36,37,38] from the structural aspect. However, multi-finger structure and closed structure have lower load capacities than ring-shaped structure. The ring-shaped structure enables the soft gripper to grasp and hold objects by radial contraction of the inner wall, such as high-load bionic winding ring-shaped soft gripper [38,39,40] and air chamber ring-shaped soft gripper [41,42,43]. The air chamber ring-shaped soft gripper with a single air chamber or multiple air chambers placed along a circle is able to achieve radial contraction under pressurization. Yu Dang et al. investigated the deformation of ring-shaped actuators with different geometrical parameter sets [36]. Zhongkui Wang et al. compared lifting force and twisting torque of three circular shell grippers with three different soft materials [37]. Ryman Hashem et al. proposed a novel bellows-driven soft pneumatic actuator with self-sensing capability [44]. However, the effect of the number of air chambers on the grasping performance (grasping range and load capacity) of the ring-shaped soft gripper has not been studied.

Inspired by biology, in this article, we present three multi-chamber ring-shaped soft grippers: a two-chamber ring-shaped soft gripper, a three-chamber ring-shaped soft gripper, and a four-chamber ring-shaped soft gripper. These ring-shaped soft grippers have the same inner wall surface area of the air chamber. These ring-shaped soft grippers are fabricated using the casting molding method. FEM models and experimental measurements are constructed to predict and compare the deformation of the three ring-shaped soft grippers. Meanwhile, FEM models are performed to predict the contact force of the three-chamber ring-shaped soft gripper, and the experimental measurements validate FEM models. In addition, several groups of load capacity experiments and application experiments are carried out to evaluate and compare the load capacity of the three ring-shaped soft grippers. The results of the above study demonstrate that the three-chamber ring-shaped soft gripper has the best grasping performance in this study.

## 2. Materials and Methods

### 2.1. Design and Structure

**Figure 1:**
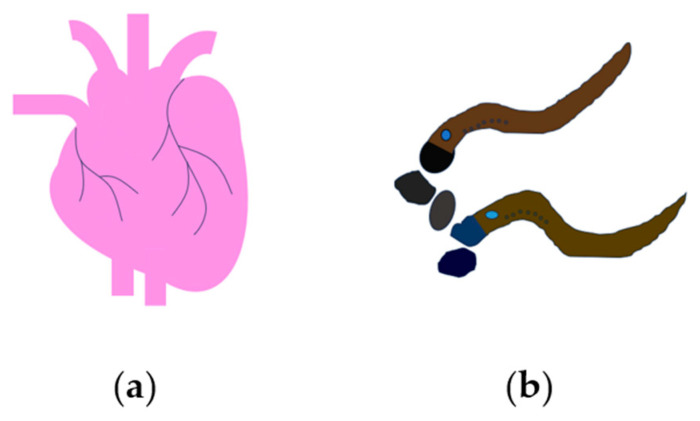
(**a**) Contraction deformation of the human or animal heart. (**b**) Stone-seizing behavior of lampreys.

As indicated in Figure 1, inspired by contraction deformation of the human or animal heart, and inspired by stone-seizing behavior of lampreys [45]. We designed and fabricated three ring-shaped soft grippers with different numbers of air chambers.

The inner wall surface area of the air chambers of the three ring-shaped soft grippers is designed to the same value to evaluate the effect of the number of air chambers on grasping performance. The inner wall surface area of the air chambers of each ring-shaped soft gripper can be expressed in Equation (1):

(1) s=πnαRH/180

where n is the number of air chambers, a is the angle of each air chamber, R is the radius of inner wall, H is the height of each air chamber. The geometrical parameters are listed in Table 1.

**Figure 2:**
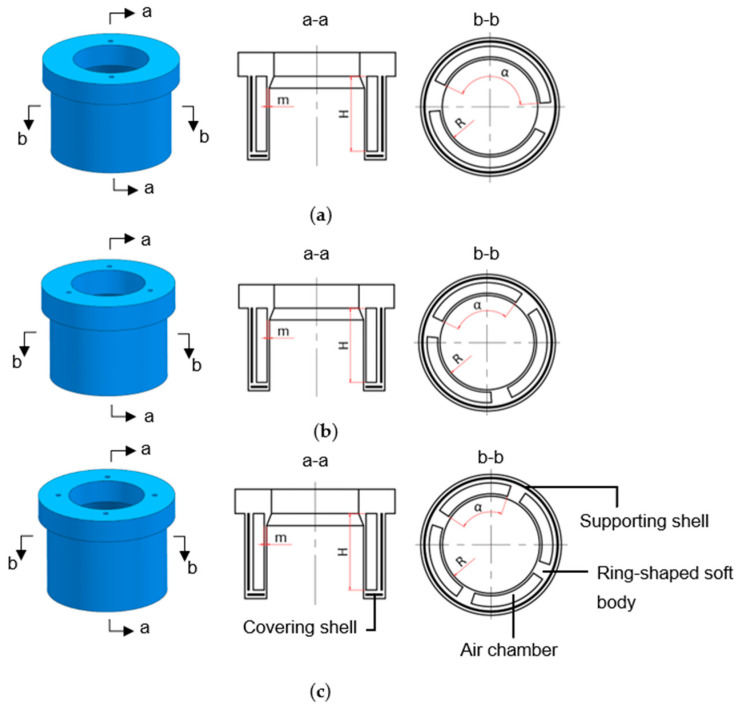
(**a**) The structure diagram of the two-chamber ring-shaped soft gripper. (**b**) The structure diagram of the three-chamber ring-shaped soft gripper. (**c**) The structure diagram of the four-chamber ring-shaped soft gripper.

As illustrated in Figure 2, the structures of the three ring-shaped soft grippers are shown respectively. Each ring-shaped soft gripper consists of a ring-shaped soft body, a supporting shell, and covering shells. Taking the four-chamber ring-shaped soft gripper as an example, we showed the position of the supporting shell and the covering shells in each ring-shaped soft gripper.

The supporting shell is embedded inside the ring-shaped soft body to constrain radial expansion of the outer wall of the ring-shaped soft gripper. The inner wall of the ring-shaped soft gripper can expand radially inward under pressurization. Each air chamber corresponds to one air hole, and each air chamber is connected with the pneumatic system through a pneumatic tube that is inserted into the air hole.

### 2.2. Fabrication

The three ring-shaped soft grippers are manufactured using the same casting molding method, the supporting shell, the covering shells, and all the molds were 3D printed with PLA material; we took the three-chamber ring-shaped soft gripper as an example to describe the fabrication process.

**Figure 3:**
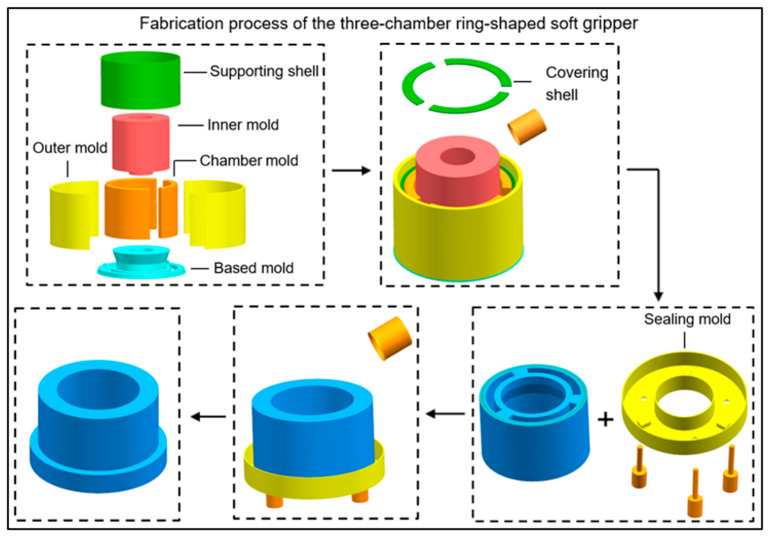
Fabrication process of the three-chamber ring-shaped soft gripper.

The fabrication process of the three-chamber ring-shaped soft gripper is shown in Figure 3. Firstly, the chamber molds, the inner mold, and the outer molds were successively fixed on the based mold. Then the supporting shell was placed in the set position of the based mold. Secondly, liquid silicon rubber material (E630, Shenzhen Hongyejie Technology Co., Ltd., Shenzhen, China) was poured into the assembled molds, forming the main function module. thirdly, the main function module was mounted on the sealing mold. Finally, liquid silicone rubber material was poured into the sealing mold and then demolded after the silicone rubber cured, forming the three-chamber ring-shaped soft gripper.

### 2.3. Material Characterization

In this study, four different hyperelastic constitutive models: Mooney-Rivlin, Ogden, Yeoh, and Arruda-Boyce were selected to fit the stress-strain data obtained from a uniaxial tensile test. The Yeoh model can better simulate the characteristics of rubber materials at large strains. The strain energy density function is defined as:

(2) $$W=\sum_{i=1}^{N}C_{i0}{\left(I_{1}-3\right)}^{i}+\sum_{k=1}^{N}\frac{1}{d_{k}}{\left(J-1\right)}^{2k}$$

where N is the order of strain energy density function, $C_{i0}$ is the material parameter, $I_{1}$ is the first strain invariant, $d_{k}$ is the material constant, and J is the volume ratio of the material after deformation to before deformation, for incompressible material, J=1.

(3) $$I_{1}=\lambda_{1}^{2}+\lambda_{2}^{2}+\lambda_{3}^{2}$$

where $\lambda_{1},{\;\lambda }_{2},and\;\lambda_{3}$ refer to the three principle stretch ratios.

We found that the second order Yeoh model in Equation (4) is in good agreement with the stress-strain data; the material parameters were obtained as $C_{10}=0.069282,C_{20}=0.001076$.

(4) $$W=C_{10}\left(I_{1}-3\right)+C_{20}{\left(I_{1}-3\right)}^{2}$$

### 2.4. Finite Element Modeling of Deformation

The deformation range of the inner wall determines the grasping range of the ring-shaped soft gripper; the larger the range of inner wall deformation, the larger the grasping range of the ring-shaped soft gripper. The grasping range is one of the important indexes of grasping performance of a ring-shaped soft gripper. Therefore, in order to predict the grasping range of the three ring-shaped soft grippers, FEM models were constructed to simulate the deformation of the inner wall of the three ring-shaped soft grippers, respectively. To simplify simulation and improve computational efficiency, the pneumatic inlets and pneumatic tubes were ignored. The surfaces of the top, bottom, and outside of the three ring-shaped soft grippers were constrained. The input air pressure varied from 0 kPa to 15 kPa at 1 kPa interval and applied on the inner surfaces of the air chambers. The contact between the inner wall was considered.

**Figure 4:**
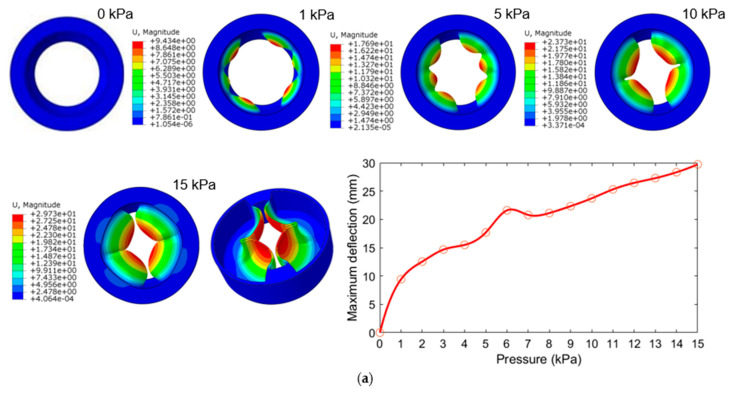

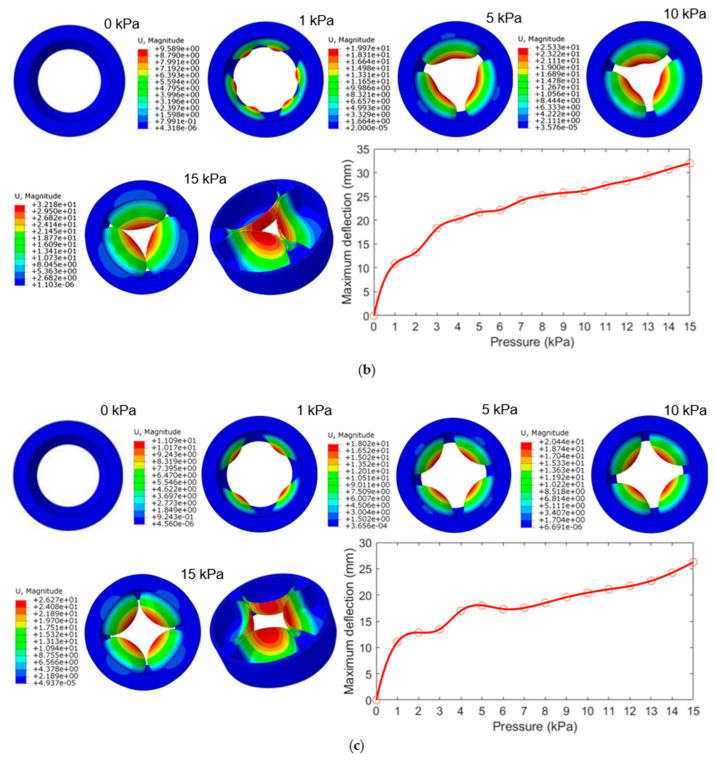
The FEM results of the deformation of the inner wall of the three ring-shaped soft grippers at prescribed input air pressures. (**a**) Two-chamber ring-shaped soft gripper. (**b**) Three-chamber ring-shaped soft gripper. (**c**) Four-chamber ring-shaped soft gripper.

As illustrated in Figure 4, the maximum deflection is used to indicate the degree of deformation of the inner wall; the deformation increases as the input air pressure increases, and the maximum deflection is at the central position of each inner wall. It is worth noting that the maximum deflection of the three-chamber ring-shaped soft gripper models (Figure 4b) is significantly larger than that of the two-chamber ring-shaped soft gripper models (Figure 4a) and the four-chamber ring-shaped soft gripper models (Figure 4c) under input air pressure from 4 kPa to 15 kPa.

### 2.5. Finite Element Modeling of Contact Force

**Figure 5:**
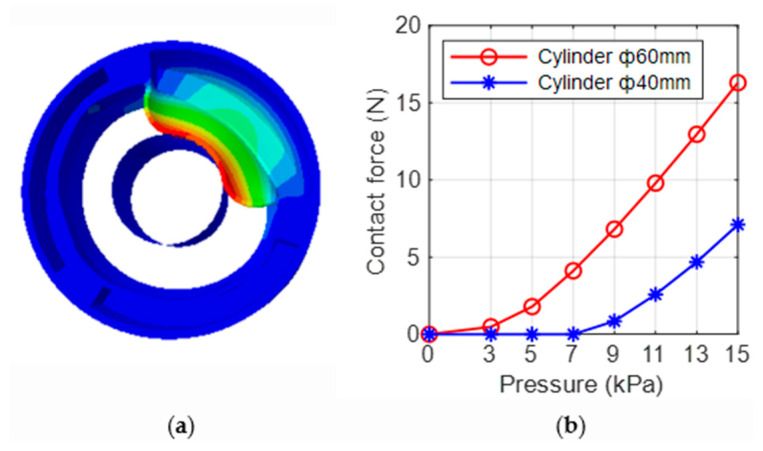
Finite element analysis of contact force. (**a**) Section-view diagram of contact force. (**b**) The relationship between contact force and input air pressures.

The inner wall of the ring-shaped soft gripper expands and deforms under input air pressure, contacting the target object and resulting in contact force. We took the three-chamber ring-shaped soft gripper as an example to study the relationship between its contact force and input air pressure. We used two cylinders (diameter 60 mm and 40 mm) as the grasping targets with PLA materials, the grasping targets were considered discrete rigid in FEM. For simplicity and intuition, the contact force between the inner wall of a single air chamber and the cylinder was analyzed (Figure 5a). The surfaces of the top, bottom, and outside of the FEM models were constrained. The finite element analysis results indicate the relationship between contact force and input air pressure, as shown in Figure 5b. As can been seen, the contact force between the inner wall and the cylinder (diameter 60 mm) is larger than that between the inner wall and the cylinder (diameter 40 mm) significantly at prescribed input air pressures; the main reason might be that the contact area between inner wall and cylinder diameter 60 mm is larger.

## 3. Results

### 3.1. Experimental Measurements of Deformation

**Figure 6:**
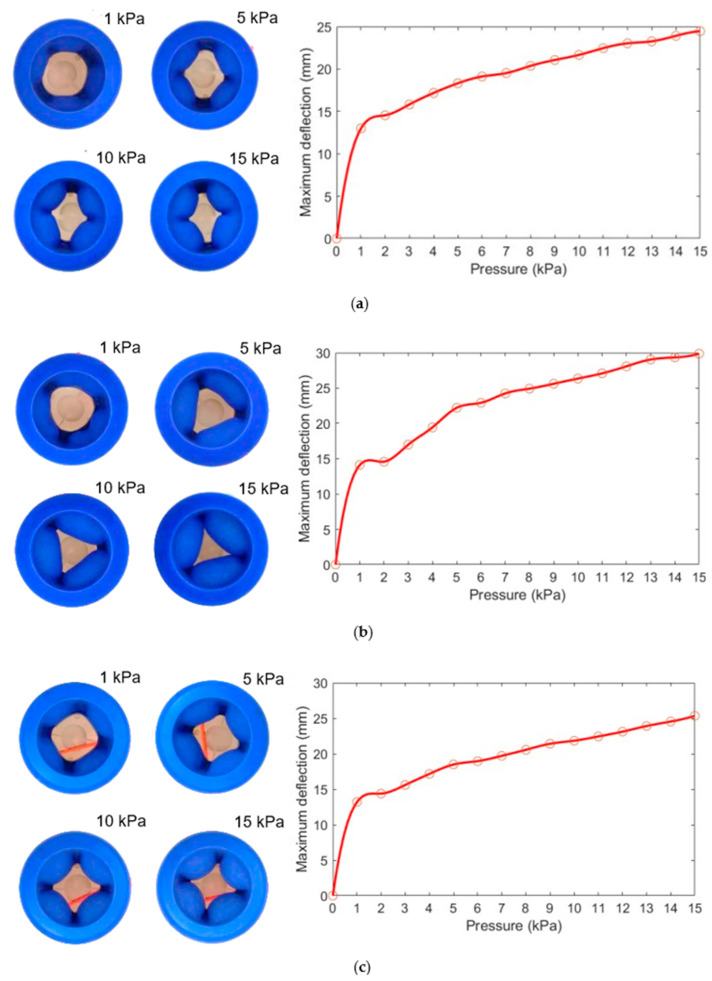
The experimental measurements of inner wall deformation of the three ring-shaped soft grippers at prescribed input pressures. (**a**) Two-chamber ring-shaped soft gripper. (**b**) Three-chamber ring-shaped soft gripper. (**c**) Four-chamber ring-shaped soft gripper.

To validate the FEM models, we measured the maximum deflection of the inner wall of the three ring-shaped soft grippers using a vernier caliper. Each inner wall was measured three times at every prescribed input air pressure, and the results were averaged. The input air pressure varied from 0 kPa to 15 kPa at 1 kPa interval. As can be seen in Figure 6, the maximum deflection of the inner wall is at its central position, and the deformation and maximum deflection increase as the input air pressure increases. After measuring and observing the deformation of the three ring-shaped soft grippers, we found that the middle position of the inner wall of the two-chamber ring-shaped soft gripper is in a concave state compared to the two sides at input air pressure from 1 kPa to 15 kPa. The middle position of the inner wall of the three-chamber ring-shaped soft gripper has a certain degree of depression compared with the two sides at input air pressure from 1 kPa to 3 kPa, moreover, the maximum deflection of the three-chamber ring-shaped soft gripper (Figure 6b) is significantly larger than that of the two-chamber ring-shaped soft gripper (Figure 6a) and the four-chamber ring-shaped soft gripper (Figure 6c) at input air pressure from 4 kPa to 15 kPa, which indicates that the grasping range of the three-chamber ring-shaped soft gripper is larger than that of other two ring-shaped soft grippers. The possible reasons might be that: (1) the angle of air chamber of the two-chamber ring-shaped soft gripper is too large, thus the arc of the corresponding inner wall is also too long, and the middle position of the inner wall is in a concave state compared to the two sides under input air pressure, which causes the inner wall to not expand sufficiently and limits the deformation of inner wall. (2) The angle of the air chamber of the four-chamber ring-shaped soft gripper is too small, thus the arc of the corresponding inner wall is also too short, which causes the limited deformation of the inner wall. (3) The angle of the air chamber of the three-chamber ring-shaped soft gripper is more appropriate than that of the two-chamber ring-shaped soft gripper and the four-chamber ring-shaped soft gripper, thus the arc length of the corresponding inner wall is also more appropriate than that of the two-chamber ring-shaped soft gripper and the four-chamber ring-shaped soft gripper; therefore, the inner wall can expand sufficiently under input air pressure from 4 kPa to 15 kPa, which causes the large deformation of the inner wall.

**Figure 7:**
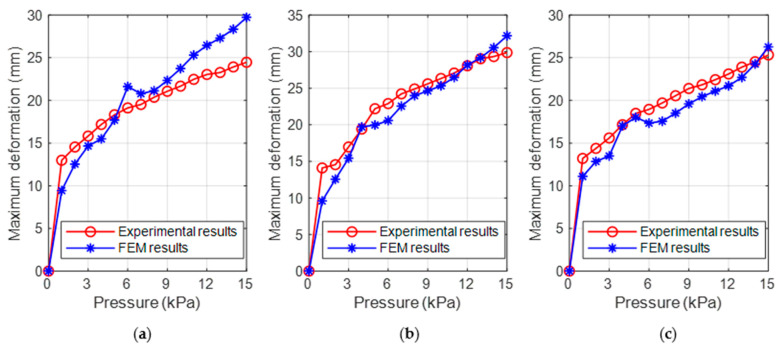
Comparison of maximum deflection of FEM results and experimental results under prescribed input air pressures. (**a**) Two-chamber ring-shaped soft gripper. (**b**) Three-chamber ring-shaped soft gripper. (**c**) Four-chamber ring-shaped soft gripper.

Figure 7 compares the maximum deflection of FEM simulation and experimental measurements of the inner wall of the three ring-shaped soft grippers at prescribed input air pressures, respectively. The red curve and the blue curve represent the experimental results and FEM results, respectively. The FEM results and experimental results show the same trend, while there are also differences between them. The most likely reason might be the simplification of the models and the complete constraint of the surfaces of the top, bottom, and outer walls in FEM; these surfaces have a small amount of deformation in experiments.

To evaluate the agreement between the FEM results and the experimental results, we calculated the relative mean deviation (Equation (5)) (ignoring the maximum deflection under 1 kPa input air pressure) of the maximum deflection; the results are 11.28% (two-chamber ring-shaped soft gripper), 5.79% (three-chamber ring-shaped soft gripper), and 6.67% (four-chamber ring-shaped soft gripper).

(5) $$\gamma =\frac{1}{14}\sum_{i=1}^{14}\frac{\left|x_{i}-\bar{x}\right|}{\bar{x}}$$

where $x_{i}$ is the maximum deflection of FEM, $\bar{x}$ is the maximum deflection of the experimental measurements.

**Figure 8:**
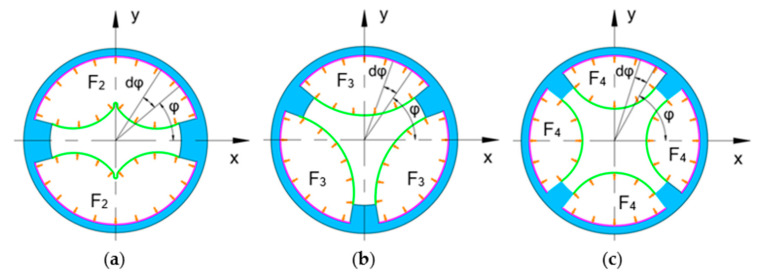
The section diagrams of the three ring-shaped soft grippers. (**a**) Two-chamber ring-shaped soft gripper. (**b**) Three-chamber ring-shaped soft gripper. (**c**) Four-chamber ring-shaped soft gripper.

The relative mean deviation of the two-chamber ring-shaped soft gripper is larger than that of the three-chamber ring-shaped soft gripper and the four-chamber ring-shaped soft gripper. The relative mean deviation of the three-chamber ring-shaped soft gripper and the four-chamber ring-shaped soft gripper is similar. The main possible reasons are shown in Figure 8. The orange arrows indicate that the air pressure acts on the inner surface of the air chamber; the green curves show the deformation of the inner wall of the ring-shaped soft gripper under input air pressure. The structure and the air pressure of each air chamber of each ring-shaped soft gripper are the same; therefore, we selected one air chamber of each ring-shaped soft gripper separately to analyze force. The air pressure acts on the inner surface of the air chamber, and the forces $F_{2}$, $F_{3}$, and $F_{4}$ are generated respectively, which causes the wall (the red curves) of the air chamber to expand and deform outward along the radial; the larger the force, the larger the deformation of each air chamber wall. In addition, manufacturing errors can also cause differences between the FEM results and the experimental results.

(6) $$F_{2}=\int_{\frac{\pi }{12}}^{\frac{11}{12}\pi }PRHd\varphi =\frac{5}{6}\pi PRH$$

(7) $$F_{3}=\int_{\frac{2}{9}\pi }^{\frac{7}{9}\pi }PRHd\varphi =\frac{5}{9}\pi PRH$$

(8) $$F_{4}=\int_{\frac{13}{45}\pi }^{\frac{127}{180}\pi }PRHd\varphi =\frac{5}{12}\pi PRH$$

where P is the air pressure, R is the inner wall radius, H is the height of air chamber.

### 3.2. Experimental Measurements of Contact Force

**Figure 9:**
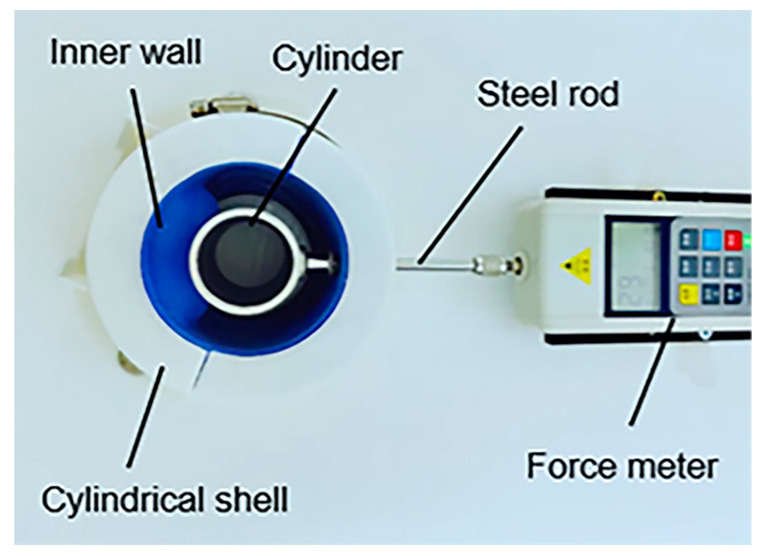
Schematic diagram of the experimental measurements of contact force.

The objective of experimental measurements of contact force were conducted to validate the FEM models. The experimental method is showed in Figure 9. First, the three-chamber ring-shaped soft gripper was installed into the cylindrical shell that constrained the surfaces of top, bottom, and outer walls of the three-chamber ring-shaped soft gripper. Second, the cylinder was placed coaxially with the three-chamber ring-shaped soft gripper on the experimental platform. Third, the cylinder and the force meter were connected at both ends of the steel rod. Then the force meter was attached to the experimental platform. Finally, the inner wall contacted the cylinder under input air pressure, and the force meter measured the contact force.

**Figure 10:**
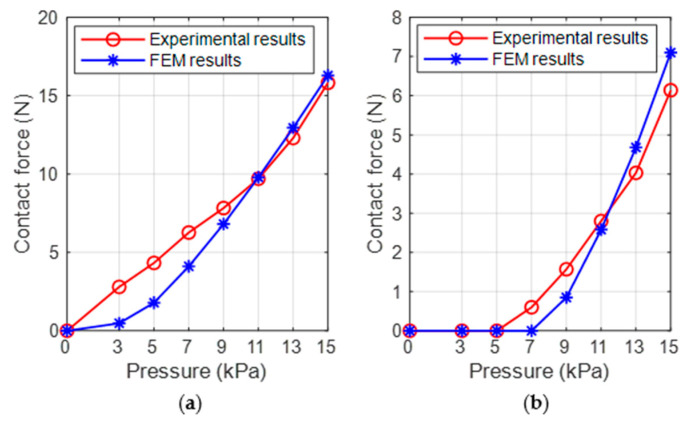
Comparison of contact force of the FEM results and experimental results under prescribed input air pressures. (**a**) Contact force between the three-chamber ring-shaped soft gripper and the cylinder diameter 60 mm. (**b**) Contact force between the three-chamber ring-shaped soft gripper and the cylinder diameter 40 mm.

The comparison between FEM analysis results and experimental measurements results are demonstrated in Figure 10. As can be seen, with the increase of input air pressure, the FEM results gradually approximate the experimental results. The results are the same for both at about 11 kPa. After that, with the increases of input air pressure, the finite element results are larger than the experimental results. The most likely reason might be as follows: the cylinder cannot maintain absolute coaxial with the three-chamber ring-shaped soft gripper. The models were simplified and the surfaces of the top, bottom, and outer walls were completely constrained in finite element analysis, while these surfaces produced a small amount of deformation in experimental measurements.

### 3.3. Experimental Measurements of Load Capacity

**Figure 11:**
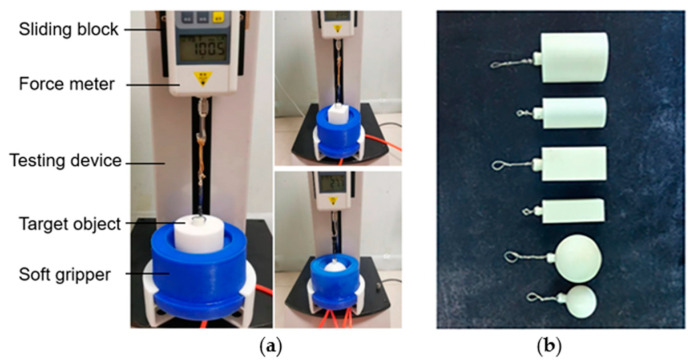
Load capacity experiments of the three ring-shaped soft grippers. (**a**) Experimental process. (**b**) Target objects.

Load capacity is one of the important indexes of grasping performance of a ring-shaped soft gripper. Several groups of experiments were conducted to evaluate the load capacity of the three ring-shaped soft grippers, as shown in Figure 11. The ring-shaped soft gripper was mounted on the testing device to grasp a target object under input air pressure (varied from 0 to 15 kPa at 1 kPa interval) (Figure 11a). These target objects were 3D printed with PLA materials (Figure 11b), including two cylinders (diameter: 40 mm and 60 mm), two spheres (diameter: 40 mm and 60 mm), and two cuboids (diameter: 40 mm and 60 mm). The force meter was fixed to the sliding block that pulled the target object upward at a fixed velocity (0.5 mm/s) until the target object was released. The force meter showed the pulling force. Each target object was tested five times at each prescribed input pressure value, and the results were averaged.

**Figure 12:**
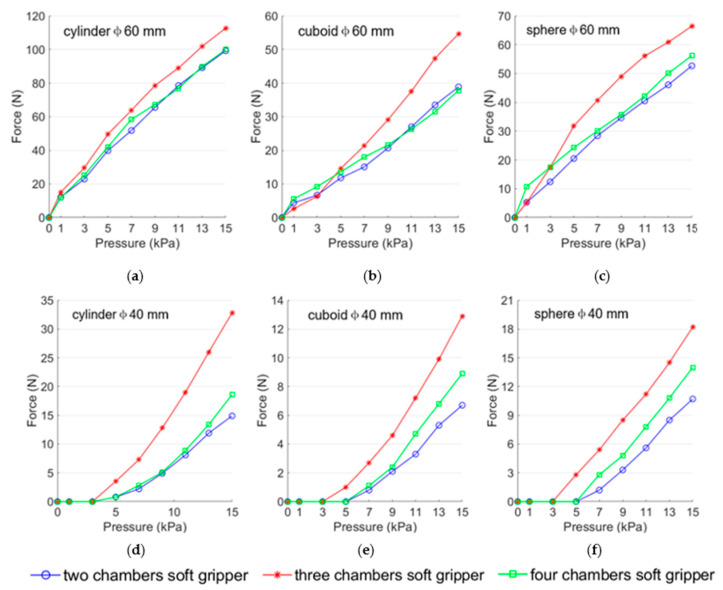
Experimental results of the load capacity of the three ring-shaped soft grippers at prescribed input air pressures. (**a**) Grasping cylinder diameter 60 mm. (**b**) Grasping cuboid diameter 60 mm. (**c**) Grasping sphere diameter 60 mm. (**d**) Grasping cylinder diameter 40 mm. (**e**) Grasping cuboid diameter 40 mm. (**f**) Grasping sphere diameter 40 mm.

The experimental results are depicted in Figure 12. In contrast, the three ring-shaped soft grippers exhibit higher load capacity when grasping the cylinders (diameter 60 mm and 40 mm) than when grasping the cuboids (diameter 60 mm and 40 mm) and spheres (diameter 60 mm and 40 mm) at prescribed air pressure; the possible reason is that the contact areas between the three ring-shaped soft grippers and the cylinders are larger than those between the three ring-shaped soft grippers and the other two target objects (the cuboids and the spheres) at prescribed air pressure. It is worth noting that the load capacity of the three-chamber ring-shaped soft gripper is higher than that of the other two ring-shaped soft grippers when grasping the same object. The most likely reason is that the contact area between the three-chamber ring-shaped soft gripper and the target object is larger than the contact area between the other two ring-shaped soft grippers and the target objects at prescribed input air pressure.

### 3.4. Practical Application

**Figure 13:**
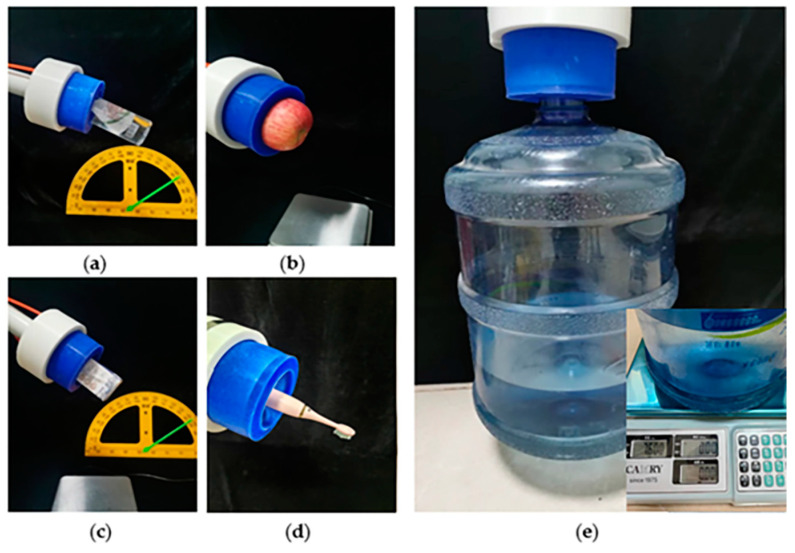
Application of the three ring-shaped soft grippers. (**a**) Picking up a bottle of water. (**b**) Grasping an apple. (**c**) Picking up a carton of milk. (**d**) Holding an electric toothbrush. (**e**) Lifting a bucket of water.

A series of application experiments were performed to validate the grasping performance and practical application value of the three ring-shaped soft grippers. As exhibited in Figure 13 and Supplementary Video S1, all three ring-shaped soft grippers can pick up a bottle of water (Ф56 mm × H210 mm, 447.9 g) (Figure 13a), grasp an apple (Ф76 mm × H72 mm, 208 g) (Figure 13b), pick up a carton of milk (L57 mm × W36 mm × H133 mm, 268.7 g) (Figure 13c), and hold an electric toothbrush (Ф25 mm × H207 mm, 65.2 g) (Figure 13d). Notably, the two-chamber ring-shaped soft gripper, the three-chamber ring-shaped soft gripper, and the four-chamber ring-shaped soft gripper can lift a bucket of water (the size of the grasped position Ф56 mm × H76 mm) with a maximum weight of 5.8 kg, 7.6 kg, and 7.5 kg, respectively (without sliding down in 20 s) (Figure 13e).

The experimental results demonstrate that the three ring-shaped soft grippers are able to grasp and manipulate a variety of objects with different weights, material properties, and shapes without complex control policy, which indicates that these ring-shaped soft grippers have potential application in industrial application and life service.

## 4. Discussion and Conclusions

In this article, we proposed three ring-shaped soft grippers with the same surface area of inner walls and different numbers of air chambers to analyze and compare their grasping performance (grasping range and load capacity). In order to compare the grasping range of the three ring-shaped soft grippers, FEM analysis of the inner wall deformation and experimental measurements of inner wall deformation of the three ring-shaped soft grippers were implemented separately; the results showed that the three-chamber ring-shaped soft gripper has the largest grasping range. Meanwhile, in order to compare the load capacity of the three ring-shaped soft grippers, experimental measurements of the load capacity of the three ring-shaped soft grippers were carried out separately, and the results showed that the three-chamber ring-shaped soft gripper has the highest load capacity. As can been seen, the three-chamber ring-shaped soft gripper has the best grasping performance in this study; therefore, we took the three-chamber ring-shaped soft gripper as an example to study the relationship between its contact force and input air pressure. Furthermore, a series of application experiments were performed, and the experimental results demonstrate that the three ring-shaped soft grippers can grasp and manipulate various objects with different weights, material properties, and shapes.

In the future, the theoretical models of the relationship between deformation and input air pressure as well as load capacity and input air pressure will be established. The structure of the three-chamber ring-shaped soft gripper will be improved for achieving large grasping range, high load capacity, and grasping target diversity.

## Figures and Tables

**Table 1:** Geometrical parameters of the three ring-shaped soft grippers.

Number of Air Chamber, n	Radius of Inner Wall, R (mm)	Height of Air Chamber, H (mm)	Angle of Air Chamber, α (°)	Thickness of Inner Wall, m (mm)
**2**	40	40	150	2
**3**	40	40	100	2
**4**	40	40	75	2

## Data Availability

Data are contained within the article as figures and tables.

## Supplementary Materials

The following supporting information can be downloaded at https://www.mdpi.com/article/10.3390/biomimetics8040337/s1, Video S1: Supplementary Video S1.
